# Supplementary material for: CO2 exposure drives a rapid pH response in live adult Drosophila
Source: PLoS One. 2024 Apr 16;19(4):e0302240. doi: 10.1371/journal.pone.0302240 (PMC11020609; doi:10.1371/journal.pone.0302240)
Supplement: S1 Text — (DOCX) [file pone.0302240.s025.docx]

library(ggplot2)

library(dplyr)

library(stats)

library(devEMF)

library(ggsignif)

library(distributions3)

library(ggpubr)

emf(file = "recovery.emf",

width = 2, height = 3,

bg = "transparent", fg = "black", pointsize = 12,

family = "arial", coordDPI = 300, emfPlus = FALSE)

df <- read.csv("S1_Data.csv")

means <- tapply(df$minutes, df$genotype,mean)

means

pvals <- compare_means(minutes ~ genotype,method = "t.test", data = df)

pvals

ggplot(df, aes(x=genotype, y=minutes, color=factor(genotype))) +

scale_color_manual(values=c('blue','magenta')) +

geom_point(position=position_jitterdodge(dodge.width=0.5),

size = 1.0) +

theme(panel.background = element_blank(),

# remove the vertical grid lines

panel.grid.major.x = element_blank() ,

# explicitly set the horizontal lines (or they will disappear too)

panel.grid.major.y = element_line( size=.25, color="gray" )) +

theme(legend.position = "none") +

theme( axis.line.x = element_blank(),

axis.ticks.y = element_blank(),

axis.ticks.x = element_line(color='gray')) +

scale_y_continuous(breaks=seq(0,12,by=2), limits = c(0, 8)) +

stat_summary(fun.data="mean_cl_normal",

geom="errorbar", width=0.5, col = "black", size = 0.5)

dev.off()
