# Supplementary material for: CO2 exposure drives a rapid pH response in live adult Drosophila
Source: PLoS One. 2024 Apr 16;19(4):e0302240. doi: 10.1371/journal.pone.0302240 (PMC11020609; doi:10.1371/journal.pone.0302240)
Supplement: Attachment — Submitted filename: Response to Reviewers.docx [file pone.0302240.s026.docx]

We thank the reviewers for their thorough analyses, insightful comments, and helpful suggestions. We are grateful for their help in improving our paper.

Below we state the reviewer’s concern, describe how we have addressed each issue, and provide the revised text in blue font.

Reviewer #1:

1. **Comment 1**: Is the recovery time following CO_2_ exposure determined by the length of the stimulus? It would be nice if authors carried out similar experiments with two different stimulus lengths. Comparing a trial shorter than one minute to a trial longer than one minute would convincingly establish this.

**Response**: We did not perform this experiment but we added the following text in paragraph 5 of the discussion. We discussed the requested experiments as part of a section on future studies (shown in **bold text**).

“We previously tested how different CO_2_ exposure regimes affected dorsal appendage development, including a single 1-minute pulse of 100% CO_2_, which increased defects in *Idgf-null* eggs but not in control eggs; a 1-minute pulse of 100% CO_2_ every 12 hours for 3.5 days, which increased defects in *Idgf-null* eggs but not in control eggs; and continuous 20% CO_2_ for up to eight days, which produced no increase in defects in either genotype [3]. Note that CO_2_ makes up about 0.04% of the ambient atmosphere [26]. **Similar experiments monitoring the pH response to different exposure regimes will determine whether the cells can manage to maintain pH homeostasis under lower (e.g., ≤ 20%) CO_2_ concentrations and for how long, whether the pH recovery profile is sensitive to the length of the CO_2_ stimulus, and whether prior exposure to CO_2_ at different concentrations alters the sensitivity of the pH response.** A study in mice showed that, after an initial decline in arterial pH, after three days of exposure to 10% CO_2_ pH returned to normal as a result of an increase in HCO_3_^-^ due to renal compensation [27]. Could Malpighian tubules, the *Drosophila* renal organ in insects, play a similar adaptive role in maintaining pH at lower concentrations of CO_2_? Our previous finding that dorsal appendage defects are enhanced in *Idgf-null* flies when exposed to a single 1-minute pulse of 100% CO_2_ but not after continuous exposure of 20% CO_2_ for several days suggests the possibility that some sort of adaptation could be occurring at lower CO_2_ concentrations.”

2. **Comment 2**: Does prior exposure to CO_2_ at different concentrations (low, medium, and high) alter the sensitivity of the pH response following CO_2_ exposure? In other words, does sensory adaptation play a role in modifying the sensitivity of the response?

**Response**: This idea is really interesting, since it invokes the process that occurs normally downstream of G-protein-coupled receptors, e.g., those involved in smell and taste. In our previous study in Reference 3, we tested the effect of different concentrations and exposure times on dorsal appendage development. We noted that dorsal appendage defects were not enhanced at 20% CO_2_ exposure, even after several days of constant 20% CO_2_, and this lack of enhancement could suggest adaptation. We predict, however, that these G-protein-coupled receptors are not involved. Our hypothesis is that CO_2_ simply enters through the spiracles and into the tracheal system, then diffuses into the hemolymph and across cell membranes of the muscle sheath and follicle cells of the ovary. Thus, CO_2_ affects pH by entering cells passively rather than by stimulating a G-protein coupled receptor. The flies used in these experiments were never exposed to CO_2_ prior to the experiments, and we did not test whether previous CO_2_ exposure at different concentrations would alter the pH response due to sensory adaptation. We added the following text to paragraph 5 of the discussion to address possible adaptation:

“A study in mice showed that, after an initial decline in arterial pH after three days of exposure to 10% CO_2_, pH returned to normal as a result of an increase in HCO3^-^ due to renal compensation [27]. Could Malpighian tubules, the *Drosophila* renal organ in insects, play a similar adaptive role in maintaining pH at lower concentrations of CO_2_? Our previous finding that dorsal appendage defects are enhanced in *Idgf-null* flies when exposed to a single1-minute pulse of 100% CO_2_ but not after continuous exposure of 20% CO_2_ for several days suggests the possibility that some sort of adaptation could be occurring at lower CO_2_ concentrations.”

3. **Comment 3**: I noticed that 100% CO_2_ has been used for imaging. I request the authors show some dose response by varying the concentration of the CO_2_ pulse.

**Response**: Varying the CO_2_ concentration would require special tanks with the ratios needed for exposing flies to precise concentrations of CO_2_. We did not perform this experiment but we added text to paragraph 5 of the discussion to address it as a possible future study (see the **bold text** in the response to Comment 1).

4. **Comment** 4: I request that the authors provide details on how the duration of the stimulus was precisely maintained in the experiment. What flow rate was used for the experiment?

**Response**: The flow rate and how it was controlled is described in the Methods section under “Fabrication of CO_2_ chamber”.

We added a sentence explaining how the timing was controlled:

“CO_2_ was supplied from a CO_2_ tank connected to a FlowBuddy^TM^ (The Flowbuddy^TM^, Genesee Scientific, cat. no. 59-122B), for finer regulation of the CO_2_ flow (approximately 3.5 liters/minute). Timing was controlled using the manual on/off switch on the FlowBuddy^TM^ and the elapsed time displayed by the Leica software during image acquisition.”

We added a description in the Results section of how flow rate and timing were controlled:

“To sustain a CO_2_ flow rate or ~3.5 l/m into the CO_2_ chamber, we used a Flowbuddy^TM^ with a manual on/off switch and controlled the timing using the elapsed time displayed by the Leica software during image acquisition (see Methods).”

5. **Comment 5**: Although I believe that the change in pH is caused by CO_2_ exposure, I request authors repeat experiments with CO_2_ receptor mutant flies.

**Response**: This is an interesting question and we added text discussing CO_2_- and acid-sensing receptors and proposed future experiments using flies lacking these receptors. We added the following text to the discussion as paragraph 6 of the discussion:

“*Drosophila* sense CO_2_ through co-expression of gustatory receptors (Gr21a and Gr63a) in the antennae; these receptors activate specific neurons that affect behavior, i.e., attraction to CO_2_ at low concentrations and avoidance at high concentrations [28]. Also, flies sense and avoid acidity through stimulation of different antennal nerves that express Ionotropic Receptor IR64a. CO_2_ dissolved in the fluid inside the antennae can form carbonic acid and activate these acid-sensitive neurons [29]. It would be interesting to test whether the pH response is in any way altered in flies lacking these receptors. We predict, however, that the follicle cell response would not differ in flies lacking these receptors. Such a response would depend on some sort of neural stimulus that would alter follicle cell physiology. Even given that the flies see CO_2_ for a whole minute, this scenario seems unlikely. Our hypothesis is that CO_2_ simply enters through the spiracles and into the tracheal system, then diffuses into the hemolymph and across cell membranes of the muscle sheath and follicle cells of the ovary. Thus, CO_2_ affects pH by entering cells passively rather than by stimulating a G-protein coupled receptor.”

6. **Comment 6**: Please explain the mechanism that determines the recovery phase after CO_2_ exposure. How does that differ between the control and the *Idgf* mutant flies? Add comments on whether altered recovery phase is linked to any physiological consequence.

**Response**: Thank you for suggesting this change. It is an important aspect of the process that needed clarifying. We have added text to paragraph 4 of the discussion to describe potential explanations for determining the pH recovery phase and why it may differ between the two genotypes:

“The rapid drop in pH_i_ was nearly identical in the two genotypes, yet the pH_i_ in ovarian cells recovered faster in *Idgf-null* flies than in control flies. The rapid drop in pH_i_ likely results from passive diffusion of dissolved CO_2_ down a concentration gradient across the cell membranes and a reaction with water to form carbonic acid, which dissociates into hydrogen ions (H^+^) and bicarbonate ions (HCO_3_^-^). The relatively slower return to homeostasis after removal of the CO_2_, depends on transport of ions across the cell membranes and is regulated by carbonic anhydrases and several membrane pumps and transporters [reviewed in 5, 17-19] as well as pH-gated channels [24, 25]. Changes in expression or activity of these proteins could impact the rate at which pH returns to a pre-CO_2_ level, but whether Idgfs influence these activities is unknown. Future studies defining the molecular mechanisms of Idgfs should give insight into this process and explain why pH_i_ recovers more rapidly in flies lacking Idgfs.”

We commented on whether the different recovery profiles are linked to any physiological consequence, specifically, dorsal appendage development, based on our previous study [3]. We added the following revision to paragraphs 2 and 3 of the discussion:

“The defects in *Idgf-null* dorsal appendages peaked at 8 - 10 hours after the CO_2_ pulse and returned to the basal level by 27 hours [3]. The wild-type flies developed normally despite the loss of cortical actin apparent at 30 minutes post CO_2_ exposure.

Considering the sensitivity of the cytoskeleton to changes in pH_i_, we hypothesize that these cytoskeletal and morphogenetic defects are a direct result of the pH_i_ perturbation, and the *Idgf-null* flies are less robust to perturbations in pH_i_ than wild-type flies. Evidently a slower recovery to pH homeostasis in control versus *Idgf-null* ovarian cells is either not a factor in determining normal dorsal appendage development or could actually be beneficial. We propose that, rather than buffering against the pH_i_ change itself, Idgfs protect against pH_i_-induced cytoskeletal modifications by indirectly modulating the cytoskeleton, which is sensitive to pH. Future studies exploring the nature of the pH-induced cytoskeletal changes (e.g., monitoring endogenously expressed fluorescent markers for key proteins such as Idgfs, actin, and actin-modifying proteins) could shed light on this unexpected result.”

7. **Comment 7**: I request authors to comment on the feasibility of using this pH sensor in other tissues.

**Response**: We added a sentence to the last paragraph of the discussion citing studies that have used this pH sensor and the live imaging procedure in other tissues:

“These tools provide opportunities to explore the effects of pH dysregulation in fly ovaries as well as a variety of other tissues [20, 21].”

Reviewer 2:

1. **Comment 1**: It would be useful for the reader to know a little more about the tpHusion expression line without having to go to the original paper. I thought it might have been Gal4 driven but this is a transgene reporter expressed “ubiquitously” using the tubulin promoter. Perhaps in the “Fly Stocks” paragraph on the authors might say “The control y,w;;tpHusion stock expresses the transgene ubiquitously from a tubulin promoter (ref 20).

**Response**: We added the sentence “The control *y w; ;tpHusion* stock expresses the transgene ubiquitously from a tubulin promoter.” to the methods. This feature is also described in the Introduction and we added a clarification in the legend for figure Figure 1 that it is ubiquitously expressed.

2. **Comment 2**: Although it is not the primary point of the paper, as mentioned above, it appears that one motivation for the work was to see if alterations in intracellular pH response might be the mechanism by which *Idgf* loss enhanced sensitivity to CO_2_. The authors refer to work which has previously demonstrated that components of cytoskeleton are pH sensitive, and this sensitivity correlates with morphogenic defects associated with CO_2_ exposure. However, the mutant *Idgf* flies actually recover more quickly than wildtype, not more slowly as one might expect if the role of idgfs was to buffer against CO_2_ induced pH changes. In my view the authors should mention this conundrum, or perhaps I am missing something.

**Response**: Thank you for pointing out this feature of the results that is not intuitive. We added text to the discussion to pose possible explanations for this “conundrum”. We have evidence from our previous study [Sustar AE, et al., Genetics. 2023;223(2)] that the presence of Idgfs protects against the damage to the actin cytoskeleton caused by exposure to CO_2_. We did not intend to suggest that the role of Idgfs is to buffer the pH but rather has a role in mitigating the cytoskeletal damage caused by a pH perturbation.

We revised paragraphs 2, 3, and 4 as shown in the response to Reviewer 1, Comment 6.

In responding to reviewer comments, we have added the following references to the manuscript (note that the original Refs 22 and 23 are now Refs 28 and 29):

22.	Feingold D, Starc T, O'Donnell MJ, Nilson L, Dent JA. The orphan pentameric ligand-gated ion channel pHCl-2 is gated by pH and regulates fluid secretion in *Drosophila* Malpighian tubules. J Exp Biol. 2016;219(Pt 17):2629-2638.

23.	Schnizler K, Saeger B, Pfeffer C, Gerbaulet A, Ebbinghaus-Kintscher U, Methfessel C, et al. A novel chloride channel in *Drosophila* *melanogaster* is inhibited by protons. J Biol Chem. 2005;280(16):16254-16262.

24.	Department of Energy. <https://netl.doe.gov/coal/carbon-storage/faqs/carbon-dioxide-101>

25.	Gates KL, Howell HA, Nair A, Vohwinkel CU, Welch LC, Beitel GJ, et al. Hypercapnia impairs lung neutrophil function and increases mortality in murine *pseudomonas* pneumonia. Am J Respir Cell Mol Biol. 2013;49(5):821-828.

26.	Kwon JY, Dahanukar A, Weiss LA, Carlson JR. The molecular basis of CO_2_ reception in *Drosophila*. Proc Natl Acad Sci U S A. 2007;104(9):3574-3578.

27.	Ai M, Min S, Grosjean Y, Leblanc C, Bell R, Benton R, et al. Acid sensing by the *Drosophila* olfactory system. Nature. 2010;468(7324):691-695.
